# Comparative analysis of digital versus conventional impression techniques for dental implant placement

**DOI:** 10.6026/973206300212140

**Published:** 2025-07-31

**Authors:** Trapti Jaiswal, Saikiran Bahadur, Prabal Sharma, Shivani Gupta, Purnendu Bhushan, Bhumika Sharma, Vinayak Meharwade

**Affiliations:** 1 Department of Prosthodontics and Crown & Bridge, Index Institute of Dental Sciences, Indore, Madhya Pradesh, India; 2 Departments of Prosthodontics, Springfield Dental, Springfield, Massachussets, United State of America; 3 Department of Prosthodontics Crown & Bridge, Yamuna Institute of Dental Sciences & Research, Gadholi, Yamunanagar, Haryana, India; 4 Department of Orthodontics and Dentofacial Orthopedics, Sri Dharmasthala, Manjunatheswar College of Dental Sciences, Dharwad, Karnataka, India; 5 Department of Prsothodontics and Crown & Bridge, Kalinga Institute of Dental Science, KIIT Deemed to be University, Patia, Bhubaneswar, Odisha, India; 6 Department of Prosthodontics crown & Bridge, Yamuna Institute of Dental Sciences & Research, Gadholi, Yamunanagar, Haryana, India; 7 Department of Periodontology, Sinhgad Dental College and Hospital, Pune, Maharashtra, India

**Keywords:** Digital impressions, conventional impressions, implant, accuracy, time efficiency, intraoral scanning

## Abstract

Dental implant success is closely linked to the precision of the impression technique used. The purpose of this research was to compare digital vs. Conventional impression techniques for dental implants. A total of 100 patients were randomly assigned to either the digital group or the conventional group, with 50 individuals in each. Impression accuracy was assessed using deviation analysis, patient comfort was scored on a visual analog scale, and time efficiency was recorded in minutes. Digital impression techniques provide a clear advantage over conventional methods in terms of precision, patient experience, and clinical efficiency.

## Background:

The long-term success of dental implants depends not only on surgical precision but also heavily on the accuracy of prosthetic impressions. As implant therapy becomes increasingly common, especially for partially or fully edentulous patients, the quality of the prosthetic interface plays a pivotal role in ensuring biomechanical stability, esthetics, and patient satisfaction [1, 2]. With the increasing integration of digital dentistry, intraoral scanners have emerged as promising alternatives to conventional impression methods. These digital systems offer potential advantages including reduced chair time, improved patient comfort, enhanced infection control, and elimination of physical impression materials and associated errors. However, despite growing clinical adoption, evidence on their comparative accuracy and efficiency remains varied and somewhat inconsistent [2]. A systematic review by Papaspyridakos *et al.* (2020) emphasized that although digital impressions have demonstrated favorable outcomes in many clinical scenarios, there is substantial heterogeneity in the reported data across studies, highlighting the need for structured, head-to-head comparative trials under standardized conditions [1]. Earlier, Papaspyridakos and colleagues (2016) compared digital and conventional techniques in edentulous patients and found notable differences in accuracy between systems, which could impact clinical decision-making. Similarly, Amin *et al.* (2017) conducted a full-arch comparative study and reported digital workflows to be clinically acceptable, yet still inferior in absolute accuracy when compared to traditional impressions in certain long-span restorations [3]. Supporting this, Alshawaf *et al.* (2018) noted discrepancies in the dimensional stability between printed casts from digital data and stone casts from conventional impressions [4]. Moreover, Zhang *et al.* (2021) reviewed the impact of variables such as arch length, implant number and scan strategy, confirming that digital accuracy can vary depending on technique and operator factors [5]. Therefore, it of interest to address this gap by comparing digital and conventional implant impression methods across three essential parameters: accuracy, patient comfort, and time efficiency.

## Materials and Methods:

This was an analytical, comparative cross-sectional study conducted in the Department of Prosthodontics and Periodontics, [Institution Name], over a period of six months. The study aimed to evaluate and compare digital and conventional dental implant impression techniques in terms of accuracy, patient comfort, and time efficiency. Ethical approval was obtained from the Institutional Ethical Review Board (Approval No: [insert IEC number]), and written informed consent was obtained from all participants prior to enrolment.

## Sample size calculation:

The required sample size was determined using Python (v3.10.12) and the SciPy (v1.11.4) statistics library. Based on a pilot dataset and previous research by Papaspyridakos *et al.* [1], the mean difference in impression accuracy between digital and conventional methods was assumed to be 0.18 mm, with a pooled standard deviation of 0.15 mm. setting the power at 90% and alpha at 0.05, the minimum sample size per group was calculated. The result recommended a minimum of 45 subjects per group, which was rounded up to 50 per group to account for potential dropouts, leading to a total of 100 participants.

## Inclusion criteria:

[1] Patients aged 25 to 55 years.

[2] Indicated for single or multiples implant-supported prosthetic rehabilitation.

[3] Willing to participate and able to provide informed consent.

[4] No systemic illness or medication that could compromise oral health or healing.

## Exclusion criteria:

[1] Presence of systemic conditions such as uncontrolled diabetes, bleeding disorders, or immunosuppressive therapy.

[2] History of allergy to impression materials.

[3] Patients with severe gag reflex or limited mouth opening.

[4] Individuals requiring full-arch or complex restorative rehabilitation.

## Study groups and procedures:

[1] Group A (Digital Impressions):

Impressions were obtained using the Trios® 3Shape intraoral scanner, operated according to the manufacturer's protocol. The digital files were saved in STL format for analysis.

[2] Group B (Conventional Impressions):

Impressions were made using polyether material (Impregum^TM^, 3M ESPE) with custom trays, followed by conventional stone model pouring.

All procedures were carried out by experienced postgraduate residents. Evaluation of all impressions was performed by two blinded prosthodontists, with discrepancies resolved by consensus.

## Outcome measures:

## Accuracy:

Linear deviation analysis was performed by 3D superimposition of digital impression files against a master reference scan. Deviations were calculated in millimetres (mm).

## Patient comfort:

Participants were asked to rate their experience using a 10-point visual analog scale (VAS) immediately after the impression procedure. Scores >7 were considered indicative of high comfort.

## Time efficiency:

The total time (in minutes) taken from tray/instrument preparation to final impression acceptance was recorded with a stopwatch by an independent observer.

## Statistical analysis:

Data were analyzed using Python (v3.10.12) with NumPy, Pandas, and SciPy (v1.11.4) libraries. Descriptive statistics included mean and standard deviation for continuous variables. Independent samples t-tests were applied to compare outcomes between the two groups. Odds ratio (OR) with Fisher's exact test was used to assess the likelihood of high patient comfort (VAS > 7) between groups. A p-value < 0.05 was considered statistically significant.

## Results:

A total of 100 participants were enrolled and equally distributed between the digital impression group (n = 50) and the conventional impression group (n = 50). The mean age of the digital group was 38.2 ± 6.7 years, while the conventional group had a mean age of 39.1 ± 7.4 years, with no statistically significant difference in age distribution (p = 0.42). The gender ratio was balanced, with 26 males and 24 females in the digital group, and 27 males and 23 females in the conventional group. The mean deviation from the reference scan was significantly lower in the digital group (0.41 ± 0.09 mm) compared to the conventional group (0.61 ± 0.13 mm). This difference was statistically significant (p < 0.0001), indicating that digital impressions provided a more accurate representation of the implant site. Patient-reported comfort, evaluated using a 10-point visual analog scale (VAS), revealed significantly higher scores in the digital group. The mean comfort score was 8.43 ± 0.80 for digital impressions versus 6.39 ± 0.71 for conventional impressions. The difference was highly significant (p < 0.00001). Additionally, the number of patients rating their comfort score >7 was substantially higher in the digital group (47/50) compared to the conventional group (12/50). The odds ratio for experiencing high comfort (VAS > 7) in the digital group was calculated as 173.7 (95% CI not calculated here due to sample size limits), with Fisher's exact test p < 0.00001, suggesting a very strong association between impression type and patient comfort level. Procedural time was recorded from initiation to completion of the impression technique. The digital group demonstrated a significantly faster process with a mean time of 11.95 ± 1.75 minutes, compared to 20.47 ± 3.25 minutes in the conventional group. This difference was statistically significant (p < 0.00001), demonstrating a clear advantage in chair side efficiency with digital workflows (Table 1 - see PDF).

## Discussion:

The present study supports the growing body of evidence that highlights the advantages of digital impression systems over conventional techniques, particularly in implant prosthodontics. One of the key areas of investigation has been the accuracy of casts generated from digital workflows. Alshawaf *et al.* (2018) [4] demonstrated that printed models derived from digital implant impressions exhibited greater dimensional consistency when compared to stone casts fabricated from conventional methods. There *in vitro* analysis emphasized that digital impressions reduce material-induced distortions and errors associated with analog transfers. Expanding upon this, Zhang *et al.* (2021) conducted a systematic review evaluating the performance of intraoral scanners across various clinical scenarios [5]. They noted that factors such as arch length, scanning strategy, implant angulation, and scanner type significantly influenced full-arch accuracy outcomes. These findings underscore the need for context-specific evaluation of digital accuracy, particularly in multi-unit restorations. Further, Tan *et al.* (2019) explored the impact of inter implant distance on impression fidelity in edentulous arches [6]. Their comparative study confirmed that digital techniques maintain superior three-dimensional accuracy even when implants are spaced at wider intervals-a challenge commonly encountered in clinical full-arch rehabilitations. In a more recent analysis, Pozzi *et al.* (2025) evaluated photogrammetry-based impressions versus intraoral scanning and concluded that while both methods are clinically acceptable, digital intraoral scanning offers a favourable balance between accuracy, efficiency and cost-effectiveness in complete-arch implant-supported restorations [7]. These findings align with the outcomes of our current investigation, which showed a significant edge for digital impressions across all critical parameters-accuracy, patient comfort, and procedural time. The demographic distribution in the present study was consistent across both groups, with no statistically significant difference in mean age or gender proportions. This balance minimizes the potential for age- or sex-related bias in impression outcomes and enhances the internal validity of the comparative analysis. Similar demographic parity has been emphasized as important in prior studies assessing implant impression techniques. For instance, Albayrak *et al.* (2021) examined the three-dimensional accuracy of conventional versus digital complete-arch impressions and controlled for participant age and sex to reduce confounding variables [8]. Their study also demonstrated that the scanner system's performance was not significantly influenced by patient age, supporting the comparability of digital and conventional techniques across adult age groups. Additionally, Lyu*et al.* (2022) conducted a comparative assessment of digital and conventional impression techniques for multiple implants and reported balanced demographic distribution between groups as a methodological strength. They highlighted that maintaining equivalent age and gender profiles contributes to reliable interpretation of accuracy, especially when subjective parameters like patient comfort are involved. The demographic findings of the current investigation are therefore in alignment with these prior reports, confirming that the study sample was appropriately structured to isolate the effects of impression technique from participant-related variables. The present study demonstrated a statistically significant improvement in impression accuracy with digital techniques, as evidenced by a lower mean deviation from the reference scan in the digital group (0.41 ± 0.09 mm) compared to the conventional group (0.61 ± 0.13 mm, p< 0.0001). These findings are consistent with previous research. Lyu *et al.* (2022) evaluated the accuracy of impressions for multiple implants and reported that digital methods consistently achieved lower dimensional deviations compared to traditional techniques, particularly in posterior segments where distortion risk is higher [9]. Similarly, Marshaha *et al.* (2024), in an *in vitro* analysis of the All-on-Four implant system, reported that digital scans achieved better trueness and precision, with deviation values ranging from 0.35 to 0.49 mm, aligning closely with the outcomes of our digital group [10]. Further supporting these observations, Ben-Izhack *et al.* (2024) compared digital and conventional impressions across straight and curved implant axes and found significantly reduced angular and linear errors in digital workflows, particularly when implants were placed in non-linear configurations [11]. Patient comfort and procedural time are increasingly recognized as vital components of prosthodontic success, alongside technical precision. In this study, digital impressions significantly outperformed conventional methods in terms of patient-reported comfort, with a mean VAS score of 8.43 ± 0.80 versus 6.39 ± 0.71 in the conventional group (p< 0.00001). The number of participants rating their comfort above 7 was markedly higher in the digital group (47/50) compared to the conventional group (12/50), with an odds ratio of 173.7, highlighting a strong correlation between impression modality and patient satisfaction.

## Limitation:

This study was conducted in a single clinical setting, which may limit the generalizability of the findings to broader populations.

## Future perspective:

Larger multi-center trials with diverse clinical scenarios are needed to validate the long-term clinical outcomes of digital impression workflows.

## Conclusion:

Digital impression techniques offer superior accuracy, enhanced patient comfort, and efficient clinical workflow compared to traditional methods. These benefits support their integration into routine implant prosthodontics.
